# Circulating inflammatory and hemostatic biomarkers are associated with risk of myocardial infarction and coronary death, but not angina pectoris, in older men

**DOI:** 10.1111/j.1538-7836.2009.03574.x

**Published:** 2009-10

**Authors:** S G WANNAMETHEE, P H WHINCUP, A G SHAPER, A RUMLEY, L LENNON, G D O LOWE

**Affiliations:** * Department of Primary Care and Population Health, UCL Medical School, Royal Free CampusLondon; † Division of Community Health Sciences, St George’s University of LondonLondon; ‡ Division of Cardiovascular and Medical Sciences, University of GlasgowGlasgow, UK

**Keywords:** angina pectoris, coronary heart disease, hemostasis, inflammation, myocardial infarction

## Abstract

*Aims*:The extent to which hemostatic and inflammatory biomarkers are related to angina pectoris as compared with myocardial infarction (MI) remains uncertain. We examined the relationship between a wide range of inflammatory and hemostatic biomarkers, including markers of activated coagulation, fibrinolysis and endothelial dysfunction and viscosity, with incident myocardial infarction (MI) or coronary heart disease (CHD) death and incident angina pectoris uncomplicated by MI or CHD death in older men. *Methods*:A prospective study of 3217 men aged 60–79 years with no baseline CHD (angina or MI) and who were not on warfarin, followed up for 7 years during which there were 198 MI/CHD death cases and 220 incident uncomplicated angina cases. *Results*:Inflammatory biomarkers [C-reactive protein (CRP), interleukin-6, fibrinogen], plasma viscosity and hemostatic biomarkers [von Willebrand factor (VWF) and fibrin D-dimer] were associated with a significant increased risk of MI/CHD death but not with uncomplicated angina even after adjustment for age and conventional risk factors. Adjustment for CRP attenuated the relationships between VWF, fibrin D-dimer and plasma viscosity with MI/CHD death. Comparisons of differing associations with risk of MI/CHD deaths and uncomplicated angina were significant for the inflammatory markers (*P* < 0.05) and marginally significant for fibrin D-dimer (*P* = 0.05). In contrast, established risk factors including blood pressure and high-density lipoprotein (HDL)-cholesterol were associated with both MI/CHD death and uncomplicated angina. *Conclusion*:Circulating biomarkers of inflammation and hemostasis are associated with incident MI/CHD death but not incident angina uncomplicated by MI or CHD death in older men.

## Introduction

It is well established that inflammation plays a major role in the development and progression of atherosclerosis, including plaque rupture which initiates coronary thrombosis and myocardial infarction (MI) [1]. Activated coagulation, endothelial dysfunction and fibrinolysis have also been associated with risk of coronary heart disease (CHD) [2–13]. The term CHD covers all forms of atherosclerotic disease of the coronary arteries with MI/coronary death as the most serious manifestation and with angina (unstable or stable) as a specific symptom complex indicating myocardial ischemia. Many persons develop only stable angina, with chest pain consistently related to physical exertion or emotional stress. Many prospective studies have shown independent associations between CHD events (MI and CHD death) and C-reactive protein (CRP), interleukin-6 (IL-6), fibrinogen, von Willebrand factor (VWF), fibrin D-dimer and tissue plasminogen activator antigen (t-PA) in both middle-aged and older populations [2–13]. In contrast, information on the association of these circulating biomarkers with the risk of uncomplicated angina events is limited.

The major pathophysiological difference distinguishing MI and other acute coronary syndromes from uncomplicated stable angina pectoris is the rupture of an atherosclerotic plaque with subsequent thrombosis formation which causes acute coronary events [1,14;]. Some studies (mainly observational in nature) have addressed the question of whether the biological profile of those who develop MI/coronary death may be different from those who have uncomplicated angina [15–19]. Conventional CHD risk factors such as blood lipids, blood pressure and smoking have been shown to predict both MI and angina [20,21;]. Few prospective studies have examined whether the relationships of inflammatory and hemostatic biomarkers to angina and MI differ but there is suggestion that many of these factors related to MI including inflammatory markers, endothelial dysfunction and fibrynolytic activity are not related to angina [8–10]. However, in a recent nested case–control study of middle-aged men (PRIME Study) inflammatory but not hemostatic factors predicted stable angina [11]. Whether these findings apply to older men is not known. We hypothesized that the relationship between inflammatory and hemostatic biomarkers including CRP, IL-6 plasma viscosity and several markers of activated coagulation, fibrinolysis and endothelial dysfunction [fibrinogen; coagulation factors VII, VIII, and IX; fibrin D-dimer, t-PA antigen, VWF activated partial thromboplastin time (APTT) and activated protein C (APC) ratio] differed between older men aged 60–79 years who develop (i) incident MI or CHD death and (ii) incident stable angina, uncomplicated by MI or CHD death.

## Subjects and methods

The British Regional Heart Study is a prospective study of cardiovascular disease involving 7735 men aged 40–59 years drawn from general practice in each of 24 British towns, who were screened between 1978 and 1980 [22]. The population studied was socio-economically representative of British men but consisted almost entirely of white Europeans (> 99%). In 1998–2000, all surviving men, now aged 60–79 years (mean age 68.7 years), were invited for a 20th year follow-up examination. The men completed a questionnaire which included questions on their medical history and lifestyle behavior. They were requested to fast for a minimum of 6 h, during which time they were instructed to drink only water and to attend for measurement at a pre-specified time between 08:00 and 18:00 hours. All men were asked to provide a blood sample, collected using the Sarstedt Monovette system. Four thousand two hundred and fifty-two men (77% of survivors) attended for examination. Four thousand and eighty-eight men had at least one hemostatic/inflammatory marker measured. We further excluded men with a recall of a diagnosis of CHD (myocardial infarction or angina) (*n* = 793) and men on warfarin (*n* = 78) leaving 3217 men for analysis.

### Cardiovascular risk factors

Anthropometric measurements including body weight, height and waist circumference (WC) were carried out. Details of measurements and classification methods for smoking status, physical activity, body mass index, WC, social class, blood pressure, high-density lipoprotein (HDL)-cholesterol, triglycerides and glucose have been described [23,24;]. Men with a doctor diagnosis of diabetes or those with a fasting glucose of ≥ 7 mmol L^−1^ (WHO criteria) were considered to have prevalent diabetes.

### Hemostatic and inflammatory biomarkers

At the 20-year examination, blood was anticoagulated with K_2_ EDTA (1.5 mg mL^−1^) for measurement of plasma viscosity at 37 °C in a semi-automated capillary viscometer (Coulter Electronics, High Wycombe, UK). Blood was also anticoagulated with 0.109 m trisodium citrate (9:1 v:v) for measurement of clottable fibrinogen (Clauss method); as well as coagulation factors (F)VII, VIII and IX; activated partial thromboplastin time (APTT) and activated protein C (APC) ratio (measured by the APTT-based method) in an MDA-180 coagulometer (Organon Teknika, Cambridge, UK). Plasma levels of t-PA antigen and D-dimer were measured with enzyme-linked immunosorbent assays (ELISA) (Biopool AB, Umea, Sweden) as was VWF antigen (Dako, High Wycombe, UK). C-reactive protein (CRP) was assayed by ultra-sensitive nephelometry (Dade Behring, Milton Keynes, UK). IL-6 was assayed using a high-sensitivity ELISA (R & D Systems, Oxford, UK). The inter-assay coefficient of variation (CV) for the biomarkers ranged from < 1% (plasma viscosity) to 8.9% (IL6) and the intra-assay CV ranged from < 1% (plasma viscosity) to 8.4% (APC ratio). Inter- and intra-assay CV for IL-6 was 8.9% and 7.5%, 8.3% and 4.7% for CRP, 3.7% and 2.6% for fibrinogen and 5.2% and 4.7% for fibrin D-dimer.

### Follow-up

All men have been followed up from initial examination (1978–1980) to June 2006 for all cause mortality and cardiovascular morbidity and follow-up has been achieved for 99% of the cohort [25]. In the present analyses, all morbidity follow-up is based on follow-up from re-screening in 1998–2000 at mean age 60–79 years, a mean follow-up period of 7 years (range 6–8 years). Information on death was collected through the established ‘tagging’ procedures provided by the National Health Service registers. Fatal coronary heart disease events were defined as death with coronary heart disease (ICD codes 410–414) as the underlying code. Evidence regarding non-fatal MI and angina was obtained by reports from general practitioners supplemented by regular 2-yearly reviews of the patients’ practice records (including hospital and clinic correspondence) carried out through to the end of the study period. Diagnosis of a non-fatal MI was confirmed in accordance with World Health Organisation criteria, on the basis of two of the following (i) severe chest pain, (ii) increased levels of cardiac enzymes and (iii) electrocardiography (ECG) changes consistent with MI. Diagnosis of uncomplicated angina refers to the development of chest pain related to exertion or stress, and not superseded by the development of MI or CHD death. Men who developed both angina and MI (or CHD death) (*n* = 32) were classified as being in the latter group. Thus, the two outcome measures (uncomplicated angina and MI/CHD death) are mutually exclusive.

## Statistical methods

Analyses are based on the division of hemostatic and inflammatory markers into equal thirds. Cox’s proportional hazards model was used to assess the multivariate-adjusted relative risk for the highest third compared with the lowest third (reference group). In the adjustment, smoking [never, long term ex-smokers (> 15 years), recent ex-smokers and current smokers], social class (7 groups), physical activity (4 groups), alcohol intake (5 groups), pre-existing diabetes (yes/no) and stroke (yes/no) were fitted as categorical variables. BMI, systolic blood pressure and HDL-C were fitted as continuous variables. Tests for trends were carried out fitting the hemostatic and inflammatory markers in its original continuous form. Differences for associations between the risk factor of interest and MI/CHD deaths and uncomplicated angina were evaluated using likelihood ratio tests based on methods of competing risk survival analysis as described by Glynn and Rosner [26]. The likelihood ratio tests evaluated the hypothesis that the associations of risk factors were the same for MI/CHD deaths and uncomplicated angina. We had 90% power to detect a mean difference of 0.19 in log CRP between men with angina and men with MI.

## Results

During the mean follow-up period of 7 years, there were 198 MI or CHD death events and 220 new diagnoses of uncomplicated angina (with no MI or CHD death) in the 3217 men with no prevalent CHD baseline.

Table 1 shows the age-adjusted baseline characteristics in the men who developed angina only, those who developed MI/CHD death, and in men who remained free of CHD. Smoking, diabetes and physical inactivity were associated with MI/CHD death but not with uncomplicated angina. Total blood cholesterol was significantly higher in men with angina and was also higher in men with MI/CHD death but the difference was not statistically significant compared with those with no CHD. Systolic blood pressure, HDL-cholesterol and triglycerides were associated with both outcomes; no significant associations were seen with WC, BMI, alcohol intake or social class. Inflammatory biomarkers (CRP, IL-6, fibrinogen), plasma viscosity, endothelial dysfunction markers (VWF, t-PA), and coagulation factors (VIII and IX, but not VII) were significantly higher in cases of MI/CHD death, but not in cases of incident angina compared with those who were CHD event free. No association was seen with APTT or APC ratio.

**Table 1:** Age-adjusted distributions of baseline characteristics and age-adjusted mean levels of cardiovascular risk factors and inflammatory/hemostatic biomarkers according to CHD status at follow-up (none, angina only, or MI/CHD death)

CHD status	None (*n* = 2809)	Angina only (*n* = 220)	*P*-value for difference Comparison with none	MI/CHD death (*n* = 198)	*P*-value for difference Comparison with none
% Current smokers	12.5	12.6	0.98	21.2	0.0002
% Physically inactive	9.1	10.7	0.37	13.6	0.07
% Manual occupation	52.3	54.8	0.46	58.3	0.11
% Light/moderate drinkers	40.5	42.7	0.57	38.2	0.18
% Diabetes	10.2	11.2	0.62	19.2	0.0001
% Stroke	4.1	5.6	0.21	6.7	0.21
Waist circumference (cm)	96.9	97.5	0.57	97.3	0.59
BMI (kg m^−2^)	26.8	27.1	0.40	26.9	0.54
SBP (mmHg)	149.6	153.3	0.02	157.2	< 0.0001
Cholesterol (mmol L^−1^)	6.06	6.21	0.04	6.13	0.32
HDL-C (mmol L^−1^)	1.35	1.26	0.002	1.25	< 0.0001
Triglyceride (mmol L )	1.60	1.77	0.006	1.73	0.01
Inflammatory and hemostatic markers					
CRP (g L^−1^)	1.63	1.68	0.66	2.22	< 0.0001
IL6 (pg mL^−1^)	2.36	2.41	0.59	2.89	< 0.0001
Fibrinogen (g L^−1^)	3.23	3.23	0.94	3.42	< 0.0001
VWF (IU dL^−1^)	137.1	137.7	0.87	146.4	0.005
t-PA (ng mL^−1^)	10.81	10.95	0.65	11.45	0.05
D-dimer (ng mL^−1^)	79.8	79.8	0.91	95.6	0.002
Factor VII (IU dL^−1^)	117.7	118.3	0.77	119.6	0.31
Factor VIII (IU dL^−1^)	131.0	131.5	0.79	135.2	0.07
Factor IX (IU dL^−1^)	131.1	133.5	0.16	135.7	0.01
Plasma viscosity (mPa.s)	1.281	1.289	0.14	1.301	0.0006
APTT(s)	34.2	34.2	0.86	34.3	0.89
APC ratio	3.21	3.23	0.21	3.26	0.35

CHD, coronary heart disease; MI, myocardial infarction; BMI, body mass index; SBP, systolic blood pressure; HDL-C, high-density lipoprotein cholesterol; CRP, C-reactive protein; VWF, von Willebrand factor.

Table 2 shows the relative risk of CHD for the traditional CVD risk factors shown to be associated with either MI/CHD death or angina in age-adjusted analyses and then with additional adjustment for lifestyle factors (age, social class, smoking, physical activity, alcohol intake) and blood lipids (HDL-C, triglyceride and cholesterol) and systolic blood pressure. Blood lipids (HDL-C and cholesterol but not triglycerides) and systolic blood pressure remained significantly associated with incident angina after adjustment. Current smoking, diabetes, HDL-C and systolic blood pressure remained significantly associated with MI/CHD death after adjustment. No significant independent association was seen between physical activity or triglycerides and CHD events.

**Table 2:** Established cardiovascular risk factors and adjusted relative risk (95% CI) of developing angina versus MI/CHD death

	Angina only		MI/CHD death	
	Age-adjusted	Model 1	Age	Model 1
Physical inactivity (mod-vig/vig vs. inactive)	0.98 (0.65, 1.46)	1.23 (0.80, 1.88)	0.63 (0.40, 0.99)	0.80 (0.50, 1.28)
Current smoking	1.15 (0.74, 1.78)	1.02 (0.64, 1.64)	2.19 (1.46, 3.23)	1.89 (1.22, 2.91)
SBP (mmHg) (T3 vs. T1; ≥ 159.5 vs. < 139.4)	1.46 (1.07, 2.01)	1.43 (1.03, 1.99)	1.90 (1.32, 2.73)	1.76 (1.20, 2.57)
HDL (mmol L^−1^) (T3 vs. T1; ≥ 1.5 vs. < 1.2)	0.59 (0.22, 0.84)	0.59 (0.38, 0.93)	0.52 (0.36, 0.74)	0.57 (0.39, 0.84)
Diabetes	1.08 (0.72, 1.62)	0.89 (0.57, 1.40)	2.10 (1.49, 2.97)	1.91 (1.32, 2.77)
Cholesterol (mmol L^−1^) (T3 vs. T1; ≥ 6.5 vs. < 5.6)	1.50 (1.07, 2.10)	1.73 (1.19, 2.53)	1.19 (0.85, 1.68)	1.38 (0.93, 2.04)
Triglyceride (mmol L^−1^) (T3 vs. T1; ≥ 1.96 vs. < 1.28)	1.30 (0.93, 1.82)	0.77 (0.50, 1.17)	1.84 (1.28, 2.63)	1.32 (0.83, 2.08)

T3 vs. T1: top third vs. bottom third. Model 1: adjusted for age, social class, alcohol intake, BMI, prevalent stroke, and each of the other factors in the Table. CHD, coronary heart disease; MI, myocardial infarction.

Table 3 shows the age-adjusted relative risk of angina and MI/CHD death for men in the top third of the distribution of each inflammatory or hemostatic biomarker compared with men in the lowest third. Only factors shown to be associated with MI/CHD death in age-adjusted analyses are shown. No associations were seen in men with angina only. Inflammatory biomarkers (CRP, IL-6, fibrinogen), plasma viscosity, D-dimer and VWF (but not t-PA or factor IX) remained significantly associated with MI/CHD death after adjustment for age, smoking, BMI, physical activity, alcohol intake, systolic blood pressure, HDL-cholesterol, history of diabetes and stroke.

**Table 3:** Adjusted relative risk (top third vs. bottom third; 95% CI) for angina or MI/CHD death according to levels of inflammatory and hemostatic biomarkers

	Angina only			MI/CHD death				
	Unadjusted	Age-adjusted	*P*-value for linear trend	Unadjusted	Age-adjusted	*P*-value for linear trend	Adjusted +	*P*-value for linear trend
CRP (≥ 2.54 vs. < 1.0)	1.07 (0.77, 1.50)	1.13 (0.81, 1.58)	0.82	2.30 (1.60, 3.29)	2.04 (1.42, 2.93)	< 0.0001	1.84 (1.23, 2.77)	0.002
IL-6 (≥ 2.92 vs. < 1.77)	0.99 (0.71, 1.37)	1.06 (0.75, 1.48)	0.79	2.73 (1.89, 3.95)	2.31 (1.59, 3.36)	< 0.0001	1.88 (1.26, 2.83)	0.001
Fibrinogen (≥ 3.44 vs. < 2.93)	0.97 (0.71, 1.34)	1.04 (0.75, 1.42)	0.97	2.32 (1.64, 3.28)	2.05 (1.45, 2.90)	< 0.0001	1.82 (1.25, 2.63)	0.004
VWF (≥ 155 vs. < 115)	0.98 (0.72, 1.33)	1.06 (0.78, 1.45)	0.88	1.67 (1.19, 2.34)	1.38 (0.98, 1.95)	0.0009	1.24 (0.87, 1.76)	0.01
t-PA (≥ 12.1 vs. < 8.7)	1.05 (0.76, 1.45)	1.05 (0.76, 1.44)	0.61	1.29 (0.92, 1.82)	1.19 (0.85, 1.68)	0.02	0.92 (0.62, 1.34)	0.50
D-dimer (≥ 99 vs. < 56)	0.88 (0.64, 1.22)	0.97 (0.69, 1.37)	0.90	2.30 (1.60, 3.31)	1.74 (1.20, 2.53)	0.003	1.39 (0.94, 2.05)	0.02
Factor IX (≥ 144 vs. < 122)	1.20 (0.85, 1.68)	1.20 (0.86, 1.68)	0.26	1.49 (1.06, 2.11)	1.48 (1.05, 2.10)	0.01	1.29 (0.89, 1.86)	0.20
Plasma viscosity (≥ 1.31 vs. < 1.25)	1.18 (0.85, 1.65)	1.22 (0.87, 1.71)	0.17	2.53 (1.71, 3.74)	2.34 (1.58, 3.46)	0.01	1.64 (1.08, 2.49)	0.03

+ Adjusted for age, social class, alcohol intake, BMI, prevalent stroke, smoking, physical activity, diabetes, systolic blood pressure and HDL-C. Test for trend fitting biomarkers in its original continuous form. Missing data: CRP (*n* = 24); IL6 (*n* = 37); Fibrinogen (*n* = 11); VWF (*n* = 7); t-PA (*n* = 7); D-dimer (*n* = 10); Factor IX (*n* = 9); Plasma viscosity (*n* = 56). CRP, C-reactive protein; IL-6, interleukin 6; VWF, von Willebrand factor.

To allow the strengths of associations between MI/death and the individual risk factors to be compared, and to contrast the relationships with MI/CHD death and with angina, Table 4 shows the adjusted hazards ratio for MI/CHD death and for angina for a 1 SD increment in systolic blood pressure and HDL-C and the biomarkers shown to be independently associated with MI/CHD death in Table 3. Systolic blood pressure and HDL-C were both associated with risk of MI/CHD death and angina, in contrast to the inflammatory and hemostatic biomarkers which were only associated with MI/CHD death. Comparisons of differing association between the two endpoints showed significant/marginally significant differences in the relationships between the inflammatory biomarkers (CRP, IL-6, fibrinogen), fibrin D-dimer and MI/CHD death and uncomplicated angina. Among the inflammatory and hemostatic biomarkers the magnitude of association with MI/CHD death appeared to be greatest for CRP and IL-6. The associations between fibrin D-dimer, VWF or plasma viscosity and MI/CHD death were attenuated after adjustment for CRP (Table 4). In contrast, the association between systolic blood pressure and HDL-C with MI/CHD remained after adjustment for CRP. As IL-6 and fibrinogen both reflect markers of inflammation, adjustments were not made for CRP for these variables.

**Table 4:** Adjusted hazards ratio (95% CI) of MI/CHD death and uncomplicated angina for a standard deviation increase in selected biomarkers

	MI/CHD death				Uncomplicated angina		
	Model 1 adjustment	*P*-trend	Model 1 + CRP	*P*-trend	Model 1 adjustment	*P*-trend	*P-value for equal association
SBP	1.27 (1.10, 1.47)	0.0004	1.30 (1.12, 1.50)	0.0002	1.15 (1.00, 1.27)	0.04	0.29
HDL-C	0.79 (0.68, 0.94)	0.006	0.84 (0.70, 0.99)	0.04	0.76 (0.65)	0.008	0.53
CRP	1.25 (1.09, 1.46)	0.002	–		0.92 (0.79, 1.07)	0.27	0.008
IL-6	1.23 (1.10, 1.46)	0.002	–		0.94 (0.81, 1.10)	0.45	0.02
Fibrinogen	1.21 (1.06, 1.37)	0.004	–		0.91 (0.78, 1.06)	0.17	0.007
VWF	1.19 (1.04, 1.37)	0.01	1.10 (0.97, 1.30)	0.13	0.98 (0.83, 1.10)	0.80	0.09
D-dimer	1.18 (1.02, 1.34)	0.02	1.11 (1.02, 1.31)	0.22	0.93 (0.80, 1.07)	0.34	0.05
Plasma viscosity	1.15 (1.01, 1.30)	0.03	1.05 (0.89, 1.23)	0.57	1.005 (0.87, 1.16)	0.93	0.25

Model 1 adjusted for social class, alcohol intake, BMI, prevalent stroke, smoking, physical activity, diabetes, systolic blood pressure and HDL-Cholesterol. *P-value associated with the null hypothesis that the variable has the same association with MI/CHD death and uncomplicated angina (model 1). *P*-value obtained by log-likelihood ratio tests. CHD, coronary heart disease; MI, myocardial infarction; VWF, von Willebrand factor.

## Discussion

In this prospective study of British men aged 60–79 years without baseline evidence of CHD, we have confirmed the results of previous studies [2–13] that circulating biomarkers of inflammation, endothelial dysfunction, coagulation and fibrinolysis (CRP, IL-6, fibrinogen, plasma viscosity, VWF and fibrin D-dimer) are associated with risk of incident major CHD events (MI or CHD death), after adjustment for traditional CHD risk factors. In contrast, we have also shown that this profile of circulating biomarkers was not associated with risk of new angina pectoris, uncomplicated by MI or CHD death.

Our findings are consistent with limited published evidence from previous cross-sectional [16], case–control [17] and prospective [8–10] studies which have shown no association between inflammatory or hemostatic biomarkers with angina. However, in the recent report from the PRIME Study (a nested case–control prospective study of men aged 40–59 years), inflammatory markers were predictive of both acute coronary syndrome and stable angina but hemostatic markers in particular VWF plasma levels were higher in individuals who subsequently developed MI (fatal or non-fatal) but not in those who develop angina pectoris [11]. The differences in finding between the associations of inflammatory biomarkers and stable angina in the PRIME Study and the present study of older men may relate to the age differences in the two populations. With over 200 cases of incident uncomplicated stable angina, and 200 cases of incident MI/CHD death, among 3200 men followed for a mean of 7 years, the present study significantly and prospectively extends the previous literature comparing the associations of these different CHD outcomes with circulating levels of inflammatory and hemostatic biomarkers.

The evidence from this and previous studies suggests that circulating biomarkers of inflammation [9,16,17;], endothelial dysfunction, coagulation and fibrinolysis [8,10,15,17;] may be specifically related to acute plaque rupture and thrombosis (the pathophysiological basis of MI and CHD death) [1,14;] rather than to slowly progressive occlusive coronary atherosclerosis which (sometimes with coronary artery spasm) is the pathophysiological substrate of uncomplicated stable angina. This suggestion is consistent with evidence that suppression of inflammation [27,28;], coagulation [29] and thrombolysis [30] each plays a key role in treatment of acute coronary artery syndromes after plaque rupture. Such processes may potentially be less important in development of uncomplicated stable angina (or, in the arteries supplying the lower limb, uncomplicated intermittent claudication). Nevertheless, the observations in prospective studies that levels of several of these circulating biomarkers predict progressive atherosclerosis in the lower limb [31] suggest that these processes also contribute to progression of chronic arterial occlusion. The associations between VWF, fibrin D-dimer or plasma viscosity with MI/CHD deaths were attenuated after adjustment for CRP, suggesting that the relationship between these variables and CHD may reflect their association with the inflammatory response. No independent associations of coagulation factors (other than fibrinogen), APTT or APC ratio were observed with major CHD events.

Some conventional risk factors including systolic blood pressure and HDL-cholesterol were important risk factors for both MI/CHD death and angina in the present study, which is consistent with previous reports [20,21;]. In contrast, cigarette smoking, prevalent diabetes and physical inactivity were only associated with MI/CHD death in this cohort of older men consistent with findings reported by The Women’s Health Initiative Observational Study of postmenopausal women [19].

The strength and limitations of the present study require careful consideration. The study is limited by its restriction to predominantly white European men aged 60–79 years. However, the study population is socially representative and follow-up rates exceptionally high. Ascertainment of CHD death and MI is based on standard methods and both CHD mortality and MI incidence rates correspond closely with national data [32]. The ascertainment of angina is based on the presence of a clinical diagnosis, supplemented by investigations where appropriate. The validity of this approach, as indicated by its influence on future mortality and CHD incidence, has been previously demonstrated [33]. All the cases defined as angina in the present analyses were uncomplicated by the development of MI or CHD death, which would have been identified by the follow-up methods used. Moreover, only a very small proportion (∼ 2%) were admitted to hospital with chest pain during the entire follow-up period, so it is likely that almost all these men had chronic stable angina. Potential circadian variation in the biomarkers may have influenced the associations. However, adjustment for time of blood measurement did not alter the findings.

We conclude that in older men, circulating biomarkers of inflammation and hemostasis are related to incident MI/CHD death but not to incident uncomplicated angina. These findings support the potentially important roles of inflammation and thrombotic tendency in pathogensis of acute coronary syndromes and highlight the possible role for anti-inflammatory or antithrombotic therapy for those who are at high risk of MI/CHD death as indicated by their levels of inflammatory and hemostatic variables. These findings also have potential implications for prospective studies which use composite measures of CHD combining angina and MI as the endpoint when assessing the relationships between these biomarkers and CHD risk particularly in older men. Further prospective studies (and meta-analyses) are suggested to establish with greater confidence the specificity of these associations for CHD (and CVD) events associated with pathological or angiographic evidence of plaque rupture and superadded thrombosis.

## References

[1] Libby P, Ridker PM, Maseri A (2002). Inflammation and atherosclerosis. Circulation.

[2] Danesh J, Whincup PH, Walker M, Lennon L, Thomson A, Appleby P, Rumley A, Lowe GD (2001). Fibrin D-dimer and coronary heart disease: prospective study and meta-analysis. Circulation.

[3] Whincup P, Danesh J, Walker M, Lennon L, Thomson A, Appleby P, Rumley A, Lowe GDO (2002). Von Willebrand factor and coronary heart disease: prospective study and meta-analysis. Eur Heart J.

[4] Lowe GDO, Danesh J, Lewington S, Walker M, Lennon L, Thomson A, Rumley A, Whincup PH (2004). Tissue plasminogen activator antigen and coronary heart disease: prospective study and meta-analysis. Eur Heart J.

[5] Danesh J, Wheeler JG, Hirshfield GM, Eda S, Eiriksdottir G, Rumley A, Lowe GDO, Pepys MB, Gudnason V (2004). C-reactive protein and other circulating markers of inflammation in the prediction of coronary heart disease. N Engl J Med.

[6] Fibrinogen Studies Collaboration (2005). Plasma fibrinogen level and the risk of major cardiovascular diseases and non-vascular mortality: an individual participant meta-analysis. JAMA.

[7] Danesh J, Kaptoge S, Mann AG, Sarwar N, Wood A, Angleman S, Wensley F, Higgins JPT, Lennon L, Eiriksdottir G, Rumley A, Whincup PH, Lowe GDO, Gudnason V (2008). Long-term interleukin-6 levels and subsequent risk of coronary heart disease: two new prospective studies and a systematic review. PLoS Med.

[8] Cushman M, Lemaitre RN, Kuller LH, Psaty BM, Macy EM, Sharrett R, Tracy RP (1999). Fibrinolytic activation markers predict myocardial infarction in the elderly. The Cardiovascular Health Study. Arterioscler Thromb Vasc Biol.

[9] Luc G, Bard JM, Juhan Vague I, Ferrieres J, Evans A, Amouyel P, Arveiler D, Fruchart JC, Ducimetiere P (2003). C-reactive protein, interleukin-6, and fibrinogen as predictors of coronary heart disease: the PRIME Study. Arterioscler Thromb Vasc Biol.

[10] Morange PE, Simon C, Alessi MC, Luc G, Arveiler D, Ferrieres J, Amouyel P, Evans A, Ducimetiere P, Juhan-Vague I, on behalf of the Prime Study Group (2004). Endothelial cell markers and the risk of coronary heart disease: the Prospective Epidemiological Study of Myocardial Infarction (PRIME) study. Circulation.

[11] Empana JP, Canoui-Poitrine F, Luc G, Juhan-Vague I, Morange P, Arveiler D, Ferrieres J, Amouyel P, Bingham A, Montaye M, Ruidavets JB, Haas B, Evans A, Ducimetiere P, PRIME Study Group (2008). Contribution of novel biomarkers to incident stable angina and acute coronary syndrome: the PRIME Study. Eur Heart J.

[12] Zakai NA, Katz R, Jenny NS, Psaty BM, Reiner AP, Schwartz SM, Cushman M (2007). Inflammation and hemostasis biomarkers and cardiovascular risk in the elderly: the Cardiovascular Health Study. J Thromb Haemost.

[13] Tzoulaki I, Murray GD, Lee AJ, Rumley A, Lowe GDO, Fowkes GR (2007). Relative value of inflammatory, hemostatic, and rheological factors for incident myocardial infarction and stroke: the Edinburgh Artery Study. Circulation.

[14] Fuster V, Badimon L, Badimon JJ, Chesebro JH (1992). The pathogenesis of coronary artery disease and the acute coronary syndrome. N Engl J Med.

[15] Itakura H, Sobel BE, Boothroyd D, Leung LL, Iribarren C, Go AS, Fortmann SP, Quertermous T, Hlatky MA, for the Atherosclerosis Disease, vascular function and genetic epidemiology (ADVANCE) Study (2007). Do plasma biomarkers of coagulation and fibrinolysis differ between patients who have experienced an acute myocardial infarction versus stable exertional angina?. Am Heart J.

[16] Ford ES, Giles WH (2000). Serum C-reactive protein and fibrinogen concentrations and self-reported angina pectoris and myocardial infarction: findings from National Health and Nutrition Examination Survey III. J Clin Epidemiol.

[17] Bogaty P, Poirier P, Simard S, Boyer L, Solymoss S, Dagenais GR (2001). Biological profiles in subjects with recurrent acute coronary events compared with subjects with long-standing stable angina. Circulation.

[18] Dunder K, Lind L, Lagerqvist B, Zethelius B, Vessby B, Lithell H (2004). Cardiovascular risk factors for stable angina pectoris versus unheralded myocardial infarction. Am Heart J.

[19] Hsia J, Aragaki A, Bloch M, LaCroix A, Wallace R, for the WHI Investigators (2004). Predictors of angina pectoris versus myocardial infarction from the Women’s Health Initiative Observational Study. Am J Cardiol.

[20] Dawber TR (1980). The Framingham study. The Epidemiology of Atherosclerotic Disease.

[21] Hagman M, Wilhelmsen L, Wedel H, Pennert K (1987). Risk factors for angina pectoris in a population study of Swedish men. J Chronic Dis.

[22] Shaper AG, Pocock SJ, Walker M, Cohen NM, Wale CJ, Thomson AG (1981). British Regional Heart Study: cardiovascular risk factors in middle-aged men in 24 towns. BMJ.

[23] Wannamethee SG, Lowe GDO, Whincup PH, Rumley A, Walker M, Lennon L (2002). Physical activity and hemostatic and inflammatory variables in elderly men. Circulation.

[24] Emberson J, Whincup PH, Walker M, Thomas M, Alberti KGM (2002). Biochemical measures in a population based study: the effect of fasting duration and time of day. Ann Clin Biochem.

[25] Walker M, Shaper AG, Lennon L, Whincup PH (2000). Twenty year follow-up of a cohort study based in general practices in 24 British towns. J Public Health Med.

[26] Glyn RJ, Rosner B (2005). Comparison of risk factors for the competing risks of coronary heart disease, stroke and venous thromboembolism. Am J Epidemiol.

[27] Ridker PM, Cannon CP, Morrow D, Rifai N, Rose LM, McCabe CH, Pfeffer MA, Braunwald E, for the Pravastatin or Atorvastatin Evaluation and Infection Therapy–Thrombolysis in Myocardial Infarction 22 (PROVE IT–TIMI 22) Investigators (2005). C-reactive protein levels, outcomes after statin therapy. N Engl J Med.

[28] Pepys MB, Hirschfield GM, Tennent GA, Gallimore JR, Kahan MC, Bellotti V (2006). Targeting C-reactive protein for the treatment of cardiovascular disease. Nature.

[29] Eikelboom JW, Anand SS, Malmberg K, Weitz JL, Ginsberg JS, Yusuf S (2000). Unfractionated heparin and low-molecular-weight heparin in acute coronary syndrome without ST elevation: a meta-analysis. Lancet.

[30] Boland A, Dundar Y, Bagust A, Haycox A, Hill R, Mota RM, Walley T, Dickson R (2003). Early thrombolysis for the treatment of acute myocardial infarction: a systematic review and economic evaluation. Health Technol Assess.

[31] Tzoulaki I, Murray GD, Price JF, Smith FB, Lee AJ, Rumley A, Lowe GD, Fowkes FG (2006). Hemostatic factors, inflammatory markers and progressive peripheral atherosclerosis: the Edinburgh Artery Study. Am J Epidemiol.

[32] Allender S, Peto V, Scarborough P, Kaur A, Rayner M (2008). Coronary Heart Disease Statistics.

[33] Lampe FC, Whincup PH, Wannamethee SG, Shaper AG, Walker M, Ebrahim S (2000). The natural history of prevalent ischaemic heart disease in middle-aged men. Eur Heart J.

